# A study on the anti-osteoporosis mechanism of isopsoralen based on network pharmacology and molecular experiments

**DOI:** 10.1186/s13018-023-03689-6

**Published:** 2023-04-17

**Authors:** Jian Wang, Tianyu Chen, Xiang Li, Yu Zhang, Shuang Fu, Ruikun Huo, Yan Duan

**Affiliations:** 1 Department of Orthopedics, Inner Mongolia People’s Hospital, No. 20, Zhaowuda Road, Saihan District, Hohhot, Inner Mongolia Autonomous Region, 010017 China; 2 Department of Orthopedics, The Third Affiliated Hospital of Southern Medical University, Tianhe District, Guangzhou, 510630 Guangdong Province China; 3 Department of Orthopedics, The Second Affiliated Hospital of Inner Mongolia Medical University, Huimin District, Hohhot, Inner Mongolia Autonomous Region, 010110 China; 4 Department of Surgery, Inner Mongolia People’s Hospital, No. 20, Zhaowuda Road, Saihan District, Hohhot, Inner Mongolia Autonomous Region, 010017 China

**Keywords:** Isopsoralen, Osteoporosis, Network pharmacology, PI3K/AKT/mTOR pathway, Molecular docking

## Abstract

**Objective:**

Osteoporosis (OP) is a disease caused by multiple factors. Studies have pointed out that isopsoralen (IPRN) is one of the most effective drugs for the treatment of OP. Based on network pharmacological and molecular experimental analysis, the molecular mechanism of IPRN in osteoporosis is clarified.

**Methods:**

IPRN target genes and OP-related genes were predicted from the databases. Intersections were obtained and visualized. Gene Ontology (GO) and Kyoto Encyclopedia of Genes and Genomes (KEGG) enrichment analyses were performed on target genes, which was confirmed by experiments internal and external experiments. Molecular docking was used to verify the binding between IPRN and target proteins. Molecular dynamics (MD) simulates the binding affinity of protein targets and active compounds.

**Results:**

87 IPRN target genes and 242 disease-related targets were predicted. The protein–protein interaction (PPI) network identified 18 IPRN target proteins for the treatment of OP. GO analysis indicated that target genes were involved in biological processes. KEGG analysis showed that pathways such as PI3K/AKT/mTOR were associated with OP. Cell experiments (qPCR and WB) found that the expressions of PI3K, AKT, and mTOR in MC3T3-E1 cells at 10 μM, 20 μM, and 50 μM IPRN concentrations, especially at 20 μM IPRN treatment, were higher than those in the control group at 48 h. Animal experiments also showed that compared with the control group, 40 mg/kg/time IPRN could promote the expression of the PI3K gene in chondrocytes of SD rats.

**Conclusions:**

This study predicted the target genes of IPRN in the treatment of OP and preliminarily verified that IPRN plays an anti-OP role through the PI3K/AKT/mTOR pathway, which provides a new drug for the treatment of OP.

## Introduction

Osteoporosis (OP) is a chronic disease with impaired bone strength caused by low bone density and bone microstructure damage, which mainly occurs in adults, especially postmenopausal women [1, 2]. Since bone density and bone quality are specific manifestations of bone strength, OP will damage bone mineral density, leading to increased fragility and further fracture [3]. The causes of OP are mainly related to low estrogen content, insufficient calcium intake and vitamin D supplementation, and excessive use of glucocorticoids [4, 5]. The literature indicates that corticosteroid-induced osteoporosis (CIO) is the most common type of secondary osteoporosis, which can lead to fracture and increase morbidity and mortality [6]. Statistical research found that through meta-analysis, the prevalence of OP in countries around the world was 18.3% (95% CI: 16.2–20.7), and the prevalence of OP in Africa was the highest at 39.5% (95% CI: 22.3–59.7) [7]. In addition, it pointed out that in a cross-sectional study of 20,416 people, the prevalence of OP among Chinese adults aged 40 and above in the mainland was 5.0% in males and 20.6% in females [8]. Obviously, with such a serious aging population in China, the incidence of OP is increasing year by year. Therefore, researching the mechanism and treatment of OP has become a popular direction. At present, its specific pathogenesis and treatment are still under in-depth research.

Currently, On the one hand, the popular biochemical markers of bone turnover (BTM), such as serum bone alkaline phosphatase (bALP) and type I procollagen N propeptide (PINP), provide specific and dynamic indicators of bone turnover mechanism in the balance between bone formation and bone absorption, and can be used as predictive factors of OP occurrence and monitoring indicators of OP patients [9]. On the other hand, hormone replacement therapy (HRT) is used to treat OP in clinical practice. Raloxifene, for example, is a selective estrogen receptor modulator (SERM) for the treatment and prevention of OP. It has been shown to reduce the risk of vertebral fractures by 36% in large clinical trials. Therefore, it greatly improves estrogen's protection of bones from fractures [10]. In addition, it also includes other anti-absorption prescription drugs for OP, such as bisphosphonate (BP) (alendronate, risedronate, and so on) and RANK ligand inhibitors (such as denosumab) [11, 12]. However, clinical findings have suggested that although commonly used anti-OP drugs are effective, severe side effects and loss of potency may limit the long-term use of single drugs [13]. And the long-term use of hormone therapy in patients will increase the risk of cardiovascular disease, coronary heart disease, breast cancer, and other diseases to varying degrees [14].

**Fig. 1:**
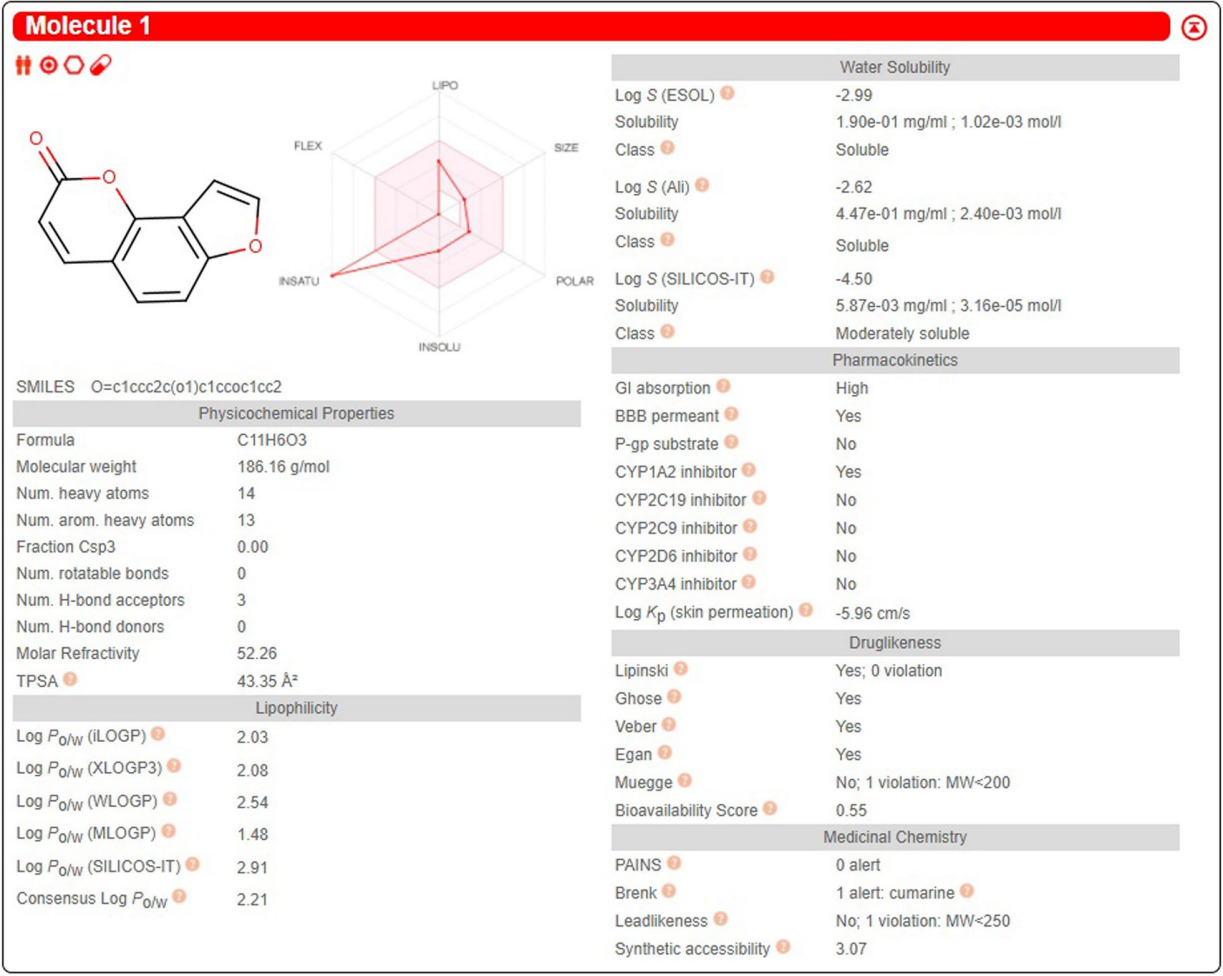
Pharmacological and molecular properties of isopspralen by SwissADME

Therefore, we try to find a suitable and effective traditional Chinese medicine (TCM), to reduce the harm of side effects to the body as much as possible. Previous studies have shown that *Epimedium* [15], *Angelica Sinensis* root [16], and Du-Zhong (*Eucommia ulmoides*) cortex extracts (DZCE) [17] in TCM can prevent or delay the onset of OP. In addition, another TCM, *Psoralea corylifolia* Linn (PCL) also plays an important role in the treatment of OP. The study has found that PCL is a kind of coumarin-type TCM compound to prevent OP, and its main active components are psoralen (PRN) and isopsoralen (IPRN) (Fig. 1) [18]. IPRN, a component of psoralen seeds isolated from previous studies, is the same as the angelica in the root of Angelica sinensis. PCL has been widely used in various TCM formulations for the treatment of cardiovascular disease, osteoporosis, cancer, and other diseases [18]. Previous literature has concluded that PRN and IPRN have biological activities such as anti-tumor [19, 20], increased levels of estrogen [21], osteoblast differentiation [22], and so on. At present, there are many studies on PRN, but few studies on the mechanism of IPRN in preventing and treating OP. Therefore, IPRN is also a good choice of TCM when studying OP caused by hormone deficiency.

At this stage, the development of network pharmacology is particularly rapid and its application is extensive. This is a new discipline based on the theory of systems biology, which analyzes the network of biological systems and selects specific signal nodes to construct a molecular network of multi-target drugs and multi-way regulation [23]. Improving the therapeutic effect of drugs and reducing toxic and side effects, thereby increasing the success rate of new drugs in clinical trials and saving the cost of drug research and development are emphasized in network pharmacology. It can be seen that the key to the modernization of TCM is to find suitable models and methods for the study of the TCM system and effective therapeutic drugs.

Therefore, based on network pharmacology, this study explored the role of IPRN in OP through different molecular targets and different pathways and was verified by cell experiments and animal experiments, aiming to provide a new direction for the treatment of OP.

## Materials and methods

### Query and visualization IPRN target genes

"Isopsoralen" was entered and searched in the PubChem (https://pubchem.ncbi.nlm.nih.gov/) Compound database, and the retrieved target genes were exported and saved in Excel. Then the STRING (https://string-db.org/) database and DAVID (https://david.ncifcrf.gov/) database were used to unify gene names and IDs [24, 25]. The obtained target genes were imported into Cytoscape 3.7.0 software to visualize the network diagram of drugs and target genes [26].

### Acquisition of OP target genes

"Osteoporosis" was sequentially entered into PubMed (https://pubmed.ncbi.nlm.nih.gov/), GeneCards (https://www.GeneCards.org/), and OMIM (Online Mendelian Inheritance in Man) (https://www.omim.org/) databases for retrieval, and the obtained genes were integrated. The duplicated genes were removed, and thus, the target-related genes of OP were finally obtained. According to the method of 2.1, Cytoscape 3.7.0 software was still used to visualize the network diagram of the disease.

### Screening of IPRN-OP intersected genes and PPI network construction and visualization

Drug target genes and OP target genes were input by Excel to make a Venn diagram. The crossover genes obtained in this section served as potential targets of IPRN to treat OP in later studies.

The common targets of IPRN-OP were input into the STRING database for retrieval. The protein type was set as "Homo sapiens", and the minimum interaction threshold was set as 0.4. Then, the network relationship data of the target interaction was obtained, and the PPI network was drawn [24]. Finally, Cytoscape software was used for visualization.

### GO annotation analysis and KEGG enrichment analysis

The Metascape platform was used to perform Gene Ontology (GO) annotation analysis and Kyoto Encyclopedia of Genes and Genomes (KEGG) pathway enrichment analysis of the targets. And the biological processes, cellular components, molecular functions, and the key signaling pathways of the targets were obtained. The analytical results were then visualized by GraphPad Prism 8.0, with *P* < 0.05 and false discovery rate (FDR) < 0.05 as the screening criteria.

### Molecular docking verification of key targets and drug components

The predicted target is evaluated by molecular docking. First, the crystal structures of candidate components and targets are obtained from the TCMSP database and RCSB protein database respectively (https://www.pdb.org/) Download. Then, AutoDockTools 1.5.6 software is used to read candidate molecules and target protein files, hydrogenate, calculate charges, add atomic types (candidate molecules as ligands, and proteins as receptors), and saves them in pdbqt format. Then, import AutoDockTools 1.5.6 to build docking grid boxes for each target. The docking is completed through Autodock Vina 1.1.2 software. The lower the vina score is, the more stable the binding between ligand and receptor is, which is used to preliminarily evaluate the binding activity of compounds to targets. Finally, Pymol 1.7.2.1 software was used to output the protein and docking results as complexes, and discovery Studio was used to analyze the force.

### Molecular dynamics simulation

To analyze the binding affinities of protein targets and active compounds obtained by molecular docking, molecular dynamics (MD) simulations were carried out by GROMACS 2020.3 software. The MD simulation workflow included four steps: energy minimization, heating, equilibrium, and production dynamics simulation. The simulation box size was optimized with the distance between each atom of the protein and the box greater than 1.0 nm. To make the simulation system electrically neutral, the water molecules were replaced with Cl^−^ and Na^+^ ions. Following the steepest descent method, energy optimization of 5.0 × 10^4^ steps was performed to minimize the energy consumption of the entire system, and finally to reduce the unreasonable contact or atom overlap in the entire system. After energy minimization, first-phase equilibration was performed with the NVT ensemble at 300 K for 100 ps to stabilize the temperature of the system. Second-phase equilibration was simulated with the NPT ensemble at 1 bar and 100 ps. The primary objective of the simulation is to optimize the interaction between the target protein and the solvent and ions so that the simulation system is fully pre-equilibrated. All MD simulations were performed for 100 ns under an isothermal and isostatic ensemble with a temperature of 300 K and a pressure of 1 atmosphere. Lennard–Jones function was used to calculate the Van der Waals force, and the nonbond truncation distance was set to 1.4 nm. The long-range electrostatic interaction was calculated by the particle Mesh-Ewald method with the Fourier spacing of 0.16 nm.

### Molecular biological verification

#### Drug-regulated cell experiments

IPRN was purchased from MCE Co., Ltd. (MedChemExpress, Monmouth Junction, NJ, USA). The product number of IPRN was HY-N0763, and its purity is 99.86%. The mouse osteoblast cell line (MC3T3-E1) was obtained from Procell Life Science&Technology Co., Ltd. (Wuhan, China).

MC3T3-E1 cells were cultured in DMEM medium containing 10% fetal bovine serum (FBS) and then were passaged and cryopreserved. The prepared MC3T3-E1 cell suspensions were divided into the control group and the experimental group. In the experimental group, three gradients of IPRN, 10 μM (low concentration), 20 μM (medium concentration), and 50 μM (high concentration), were sequentially added to the complete medium according to the conditions of the previous experiments. At 48 h after administration, cell RNA and protein samples were collected, and GAPDH was used as an internal control to detect the expression of PI3K, AKT, mTOR genes and their phosphorylated proteins by quantitative reverse transcription polymerase chain reaction (qRT-PCR) and Western blot (WB). The primer sequence is shown in Table 1.

**Table 1:** Primer sequences of GAPDH, PI3K, AKT, and mTOR genes

Gene	Primer	Sequence (5'-3')	Number of bases
GAPDH	Forward	ACTCTCCACAGTCAGACCCA	20
	Reverse	ACTCTCCACAGTCAGACCCA	20
PI3K	Forward	GGAATGTCGGGAGCAGCAACC	21
	Reverse	TCTACCACTACGGAGCAGGCATAG	24
AKT	Forward	TCAGGATGTGGATCAGCGAGAGTC	24
	Reverse	AGGCAGCGGATGATAAAGGTGTTA	24
mTOR	Forward	ACCGTCCGCCTTCACAGATACC	22
	Reverse	GCAGTCCGTTCCTTCTCCTTCTTG	24

#### Drug-regulated animal experiments

A total of 9 two-month-old Sprague–Dawley (SD) rats which were SPF grade rats, half male, and half female and weighed 200 ± 20 g, were purchased from Hubei Provincial Laboratory Animal Research Center, license number SCXK (E) 2020–0018. They were fed at the Experimental Animal Center of Xizang Minzu University. Animal experiments complied with ethics and principles.

SD rats were randomly divided into the control group and the experimental group (3 rats in each group) according to the random number table method. The control group was given the same amount of normal saline every day for 7 consecutive days. The experimental group was given IPRN (40 mg/kg/time) continuously for 7 days. At 24 h after the last gavage, the knee joint cartilage tissues of rats in the two groups were collected, and RNA was extracted. Rat β-actin was used as an internal reference, and the relative expression levels of the PI3K gene were calculated according to 2^−∆∆Ct^.

### Statistical analysis

The control group and the experimental group were repeated three times each group, and the statistical analysis was carried out in turn, and finally, the effect of IPRN on MC3T3-E1 cells and SD rats was obtained. The mean ± standard deviation (SD) of all experimental data was taken. Statistical analyses were performed by GraphPad Prism 8.0 statistical software. The data were compared between the two groups by Student’s *t* test, and *P* < 0.05 was considered to be statistically significant.

## Results

### Target search and network construction of IPRN

**Fig. 2:**
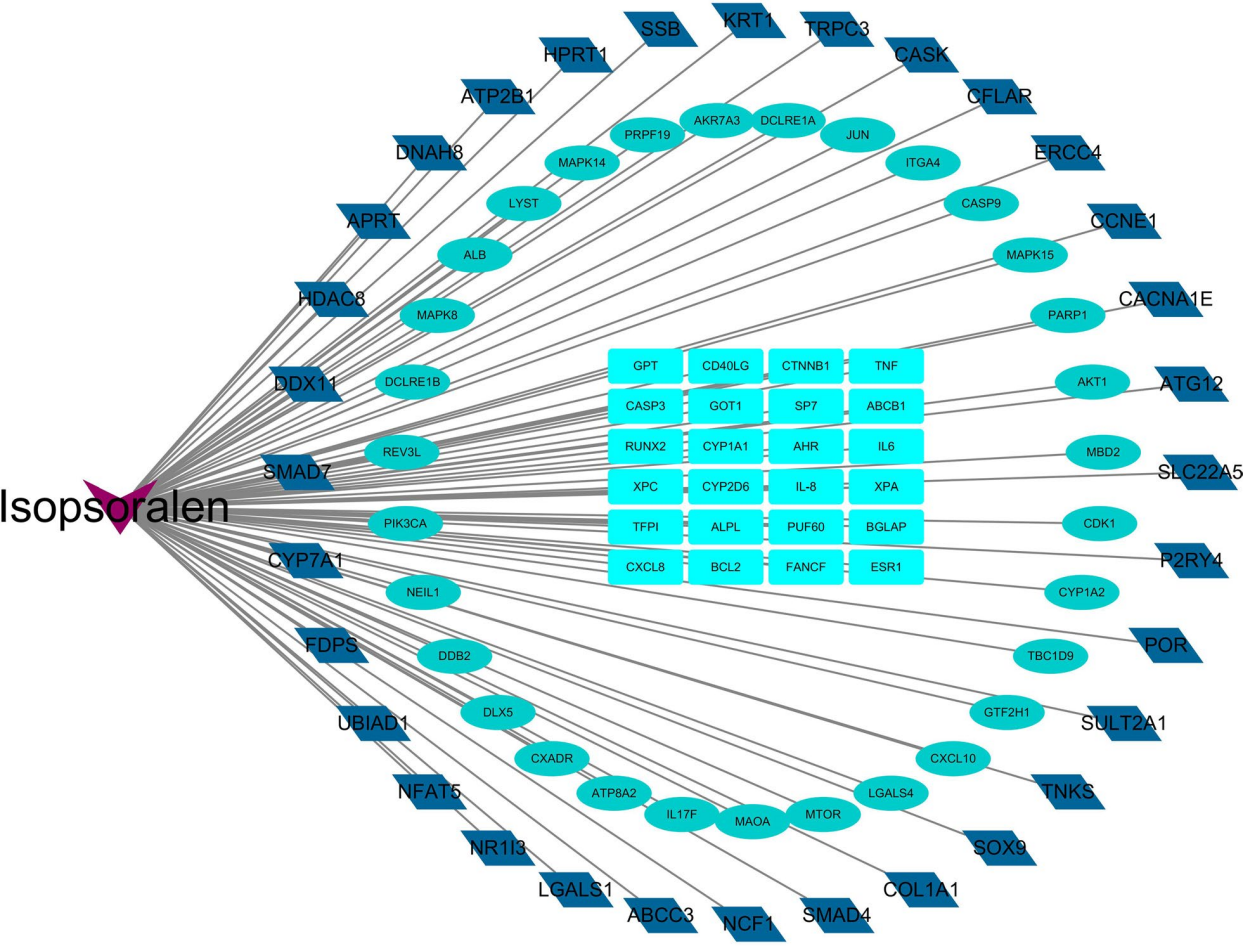
Network map of isopsoralen-related target proteins

As shown in Fig. 2, we found 87 IPRN-related target proteins and constructed a network diagram of the drug and target proteins by Cytoscape software. It can be seen from the figure that the purple inverted triangle on the left represents IPRN, and the rectangles, ellipses, and parallelograms radiating from the IPRN node represent the target proteins.

### Visualization of OP-target network

**Fig. 3:**
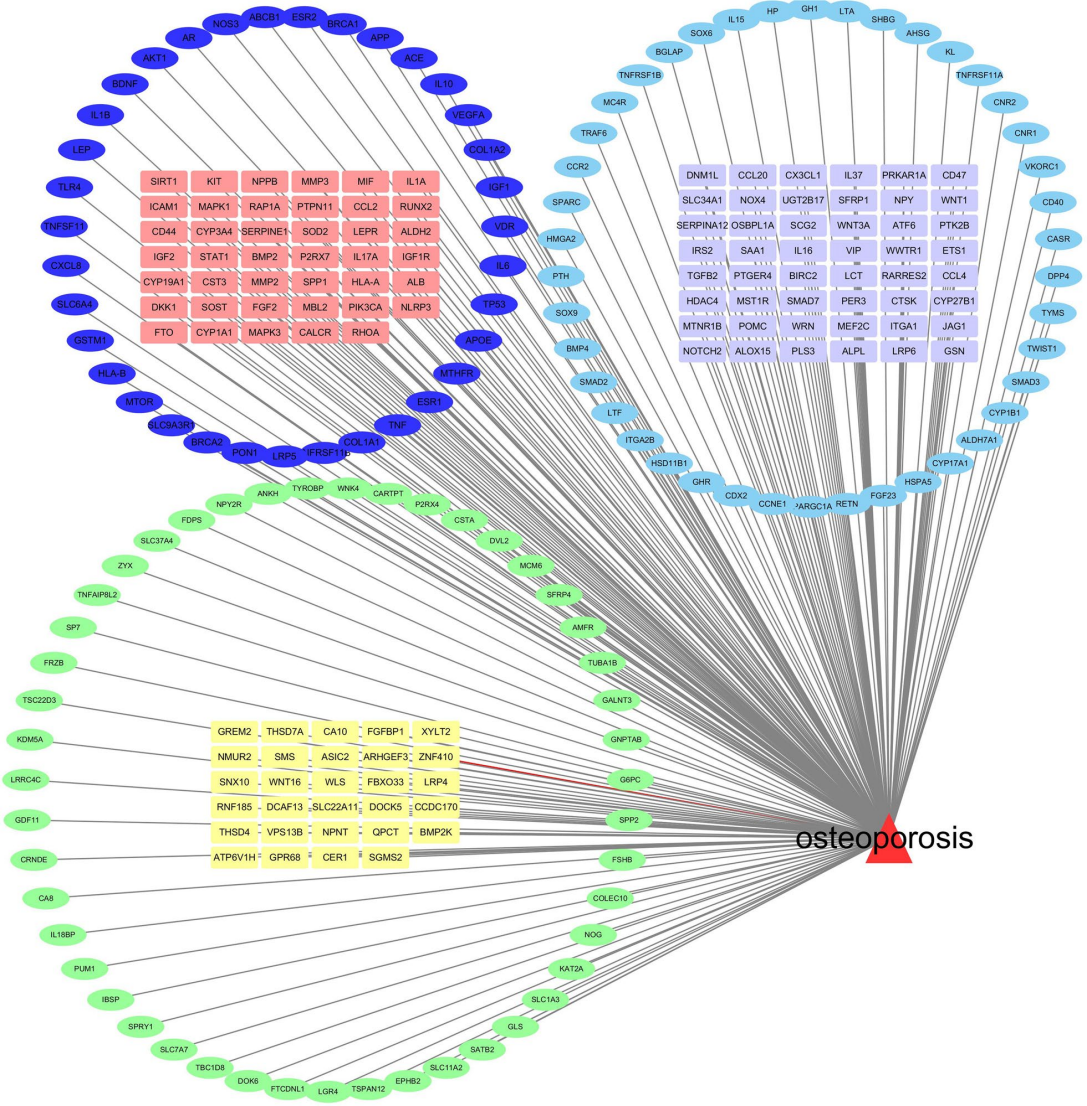
Network diagram OP targets genes

The number of OP-related genes retrieved from the PubMed, GeneCards, and OMIM databases was 317, 4066, and 34, respectively. The results from the three databases were exported and then merged in Excel to remove duplicates and take the intersected genes, and finally, 242 genes associated with OP were obtained. As shown in Fig. 3, the red triangle represents OP, and the rounded rectangles and ellipses radiating from the OP node represent OP-related genes.

### Integration of the key IPRN targets in the treatment of OP

**Fig. 4:**
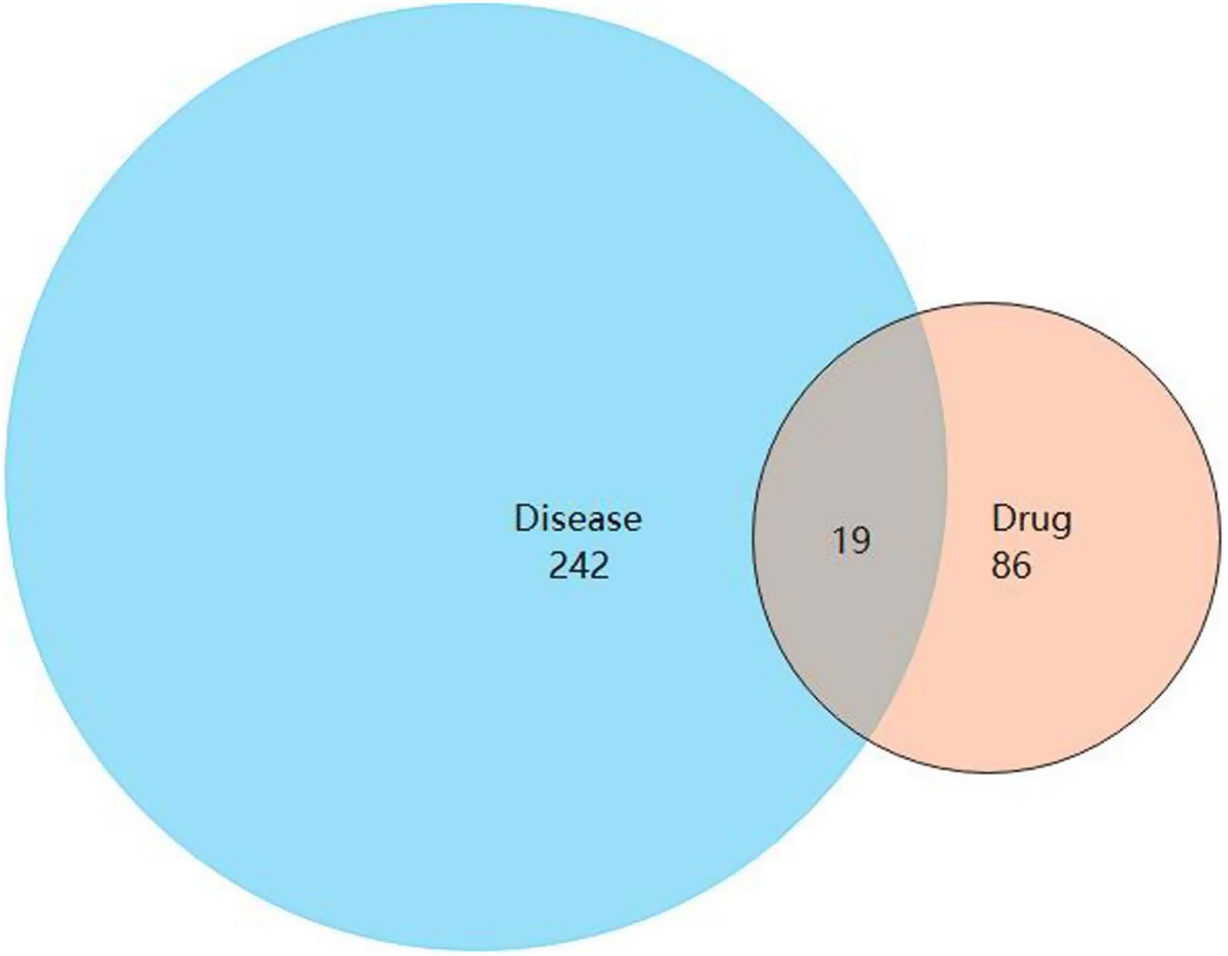
Intersection target Venn diagram

**Fig. 5:**
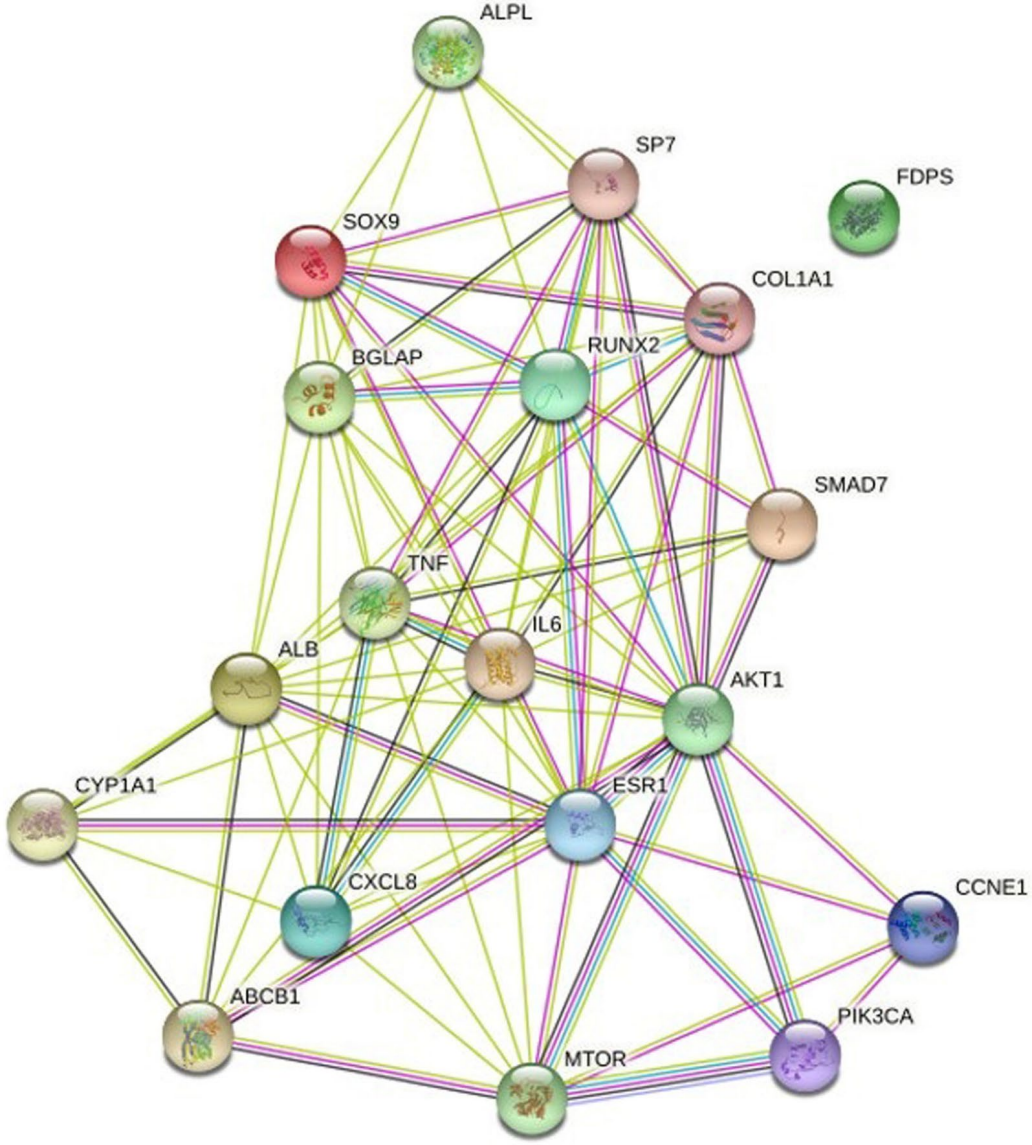
PPI network diagram of cross-targets

**Fig. 6:**
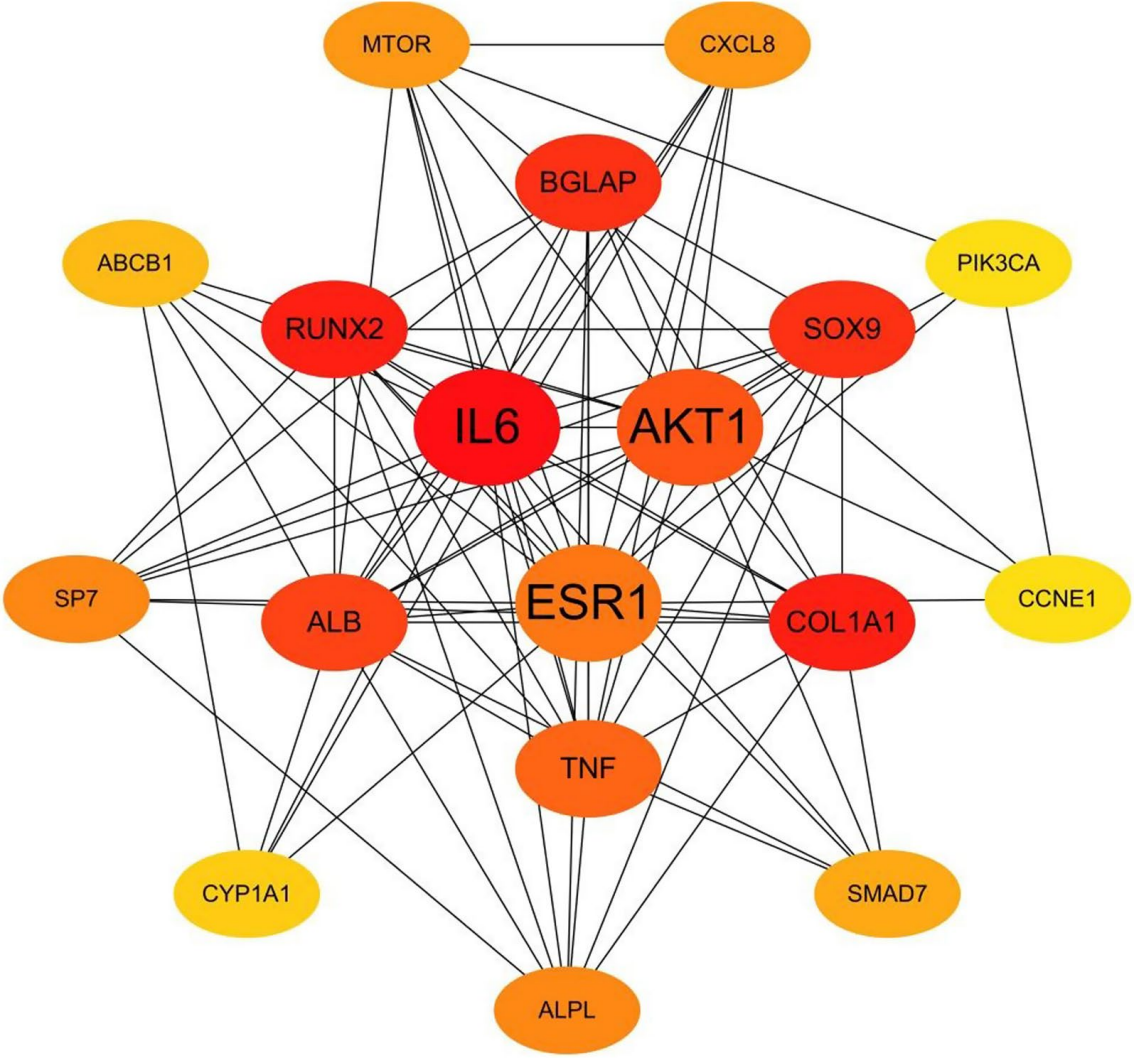
Network construction diagram of overlapping target proteins of drugs and diseases

**Fig. 7:**
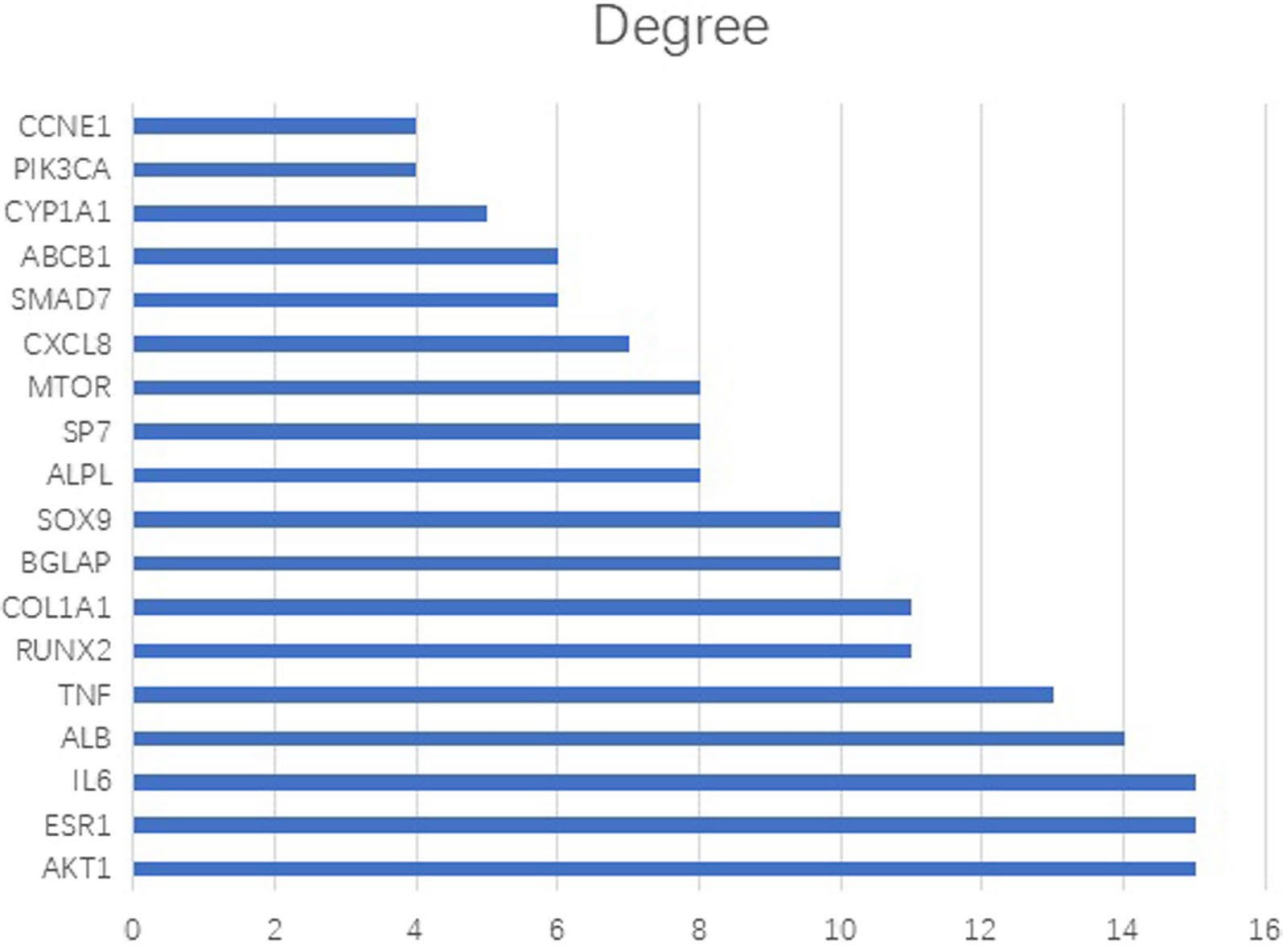
Degree values of the targets

A total of 19 common target genes were obtained from the Venn diagram (Fig. 4). These target proteins were input into the STRING online software for analysis and retrieval (Fig. 5). The target protein FDPS didn't interact with the other 18 cross-targets, so it was deleted. The results were imported into Cytoscape software for visualization (Fig. 6), and the degree values of protein interaction network nodes were analyzed in ascending order (Fig. 7). The higher the degree value reaches, the longer the histogram is, indicating that the gene is at the core of the network. As can be seen in Fig. 6, AKT serum/triamino kinase 1 (AKT1) is the core of the network. Moreover, phosphatidylinositol-4,5-bisphosphate 3-kinase catalytic subunit alpha (PIK3CA) and mechanistic target of rapamycin (mTOR) are closely related to AKT1. Therefore, AKT1, PIK3CA, and mTOR may be the key targets of IPRN in the treatment of OP, and other key overlapping target proteins are interleukin 6 (IL6), Runt-related transcription factor 2 (RUNX2), bone gamma-carboxyglutamate protein (BGLAP), SRY-box 9 (SOX9), collagen type I alpha 1 chain (COL1A1), estrogen receptor alpha (ESR1), tumor necrosis factor (TNF) and albumin (ALB). These targets may show important biological effects of IPRN in the treatment of OP. Details of the above target proteins are presented in Table 2.

**Table 2:** Basic information on overlapping target proteins of drugs and diseases

Number	Gene Symbol	Gene ID	Protein Name
1	ABCB1	5243	ATP binding cassette subfamily B member 1 (ABCB1)
1	ALB	213	Albumin (ALB)
1	CYP1A1	1543	Cytochrome p450 family 1 subfamily a member 1 (CYP1A1)
1	IL6	3569	Interleukin 6 (IL6)
1	TNF	7124	Tumor necrosis factor (TNF)
1	ESR1	2099	Estrogen receptor alpha (ESR1)
1	AKT1	207	AKT serine/threonine kinase 1 (AKT1)
1	COL1A1	1277	Collagen type I alpha 1 chain (COL1A1)
1	SOX9	6662	SRY-box 9 (SOX9)
1	SMAD7	4092	SMAD family member 7 (SMAD7)
1	CCNE1	898	Cyclin E1 (CCNE1)
1	PIK3CA	5290	Phosphatidylinositol-4,5-bisphosphate 3-kinase catalytic subunit alpha (PIK3CA)
1	CXCL8	3576	C-X-C motif chemokine ligand 8 (CXCL8)
1	MTOR	2475	Mechanistic target of rapamycin (MTOR)
1	BGLAP	632	Bone gamma-carboxyglutamate protein (BGLAP)
1	RUNX2	860	Runt-related transcription factor 2 (RUNX2)
1	SP7	121,340	Sp7 transcription factor (SP7)
1	ALPL	249	Alkaline phosphatase, liver/bone/kidney (ALPL)

### GO functional annotation scores and KEGG enrichment analysis of common targets

**Fig. 8:**
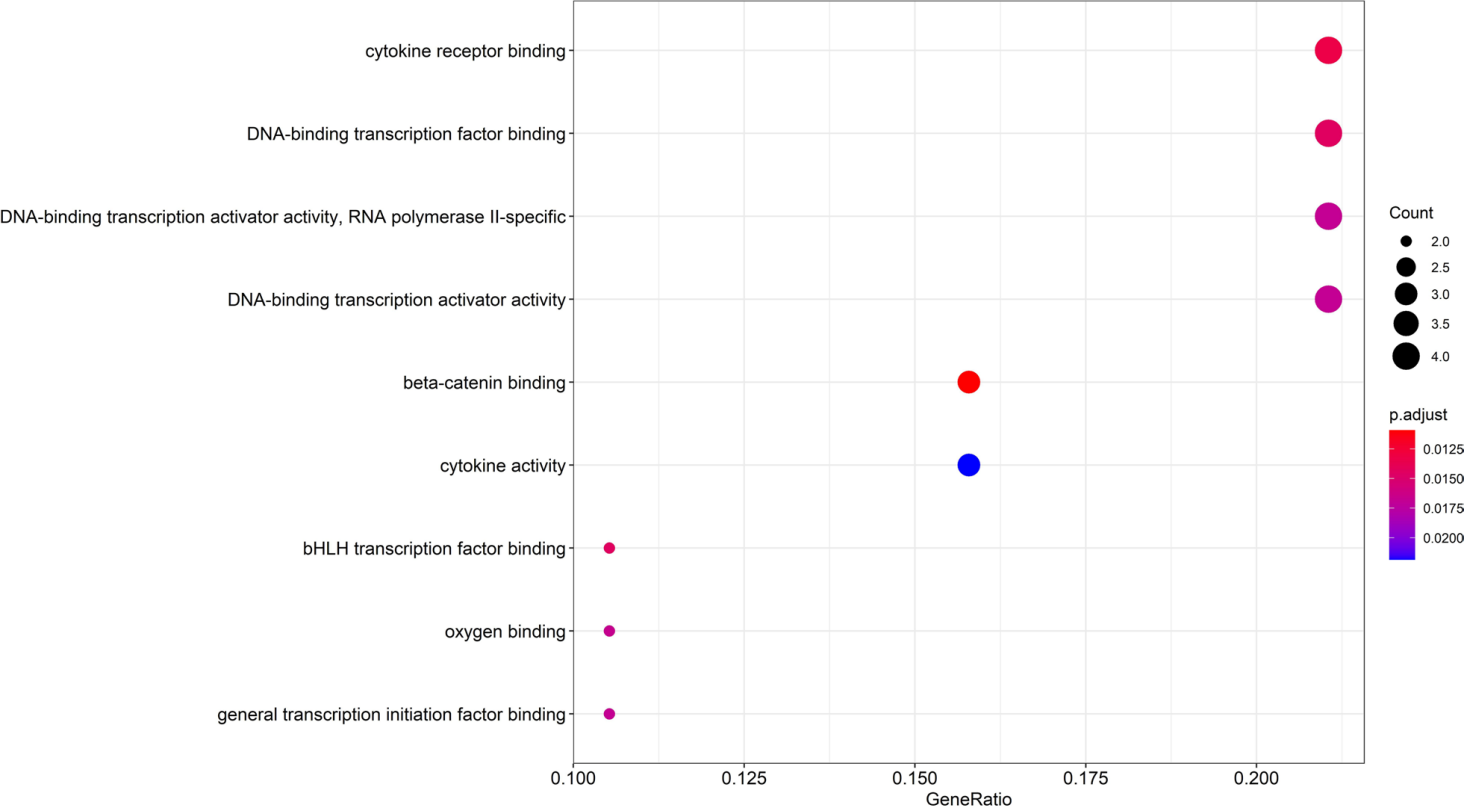
GO enrichment analysis of isopsoralen

As shown in Fig. 8, the functional distribution of 18 target proteins was explored by GO functional analysis. The GO analytical results of the predicted key IPRN targets for OP were shown in this figure. The results of this study showed that these targets can interact with cytokine receptors, β-catenin, DNA-binding transcription factors, cell proliferation, etc. These findings further indicated that IPRN may cause OP by participating in these biological processes.

**Fig. 9:**
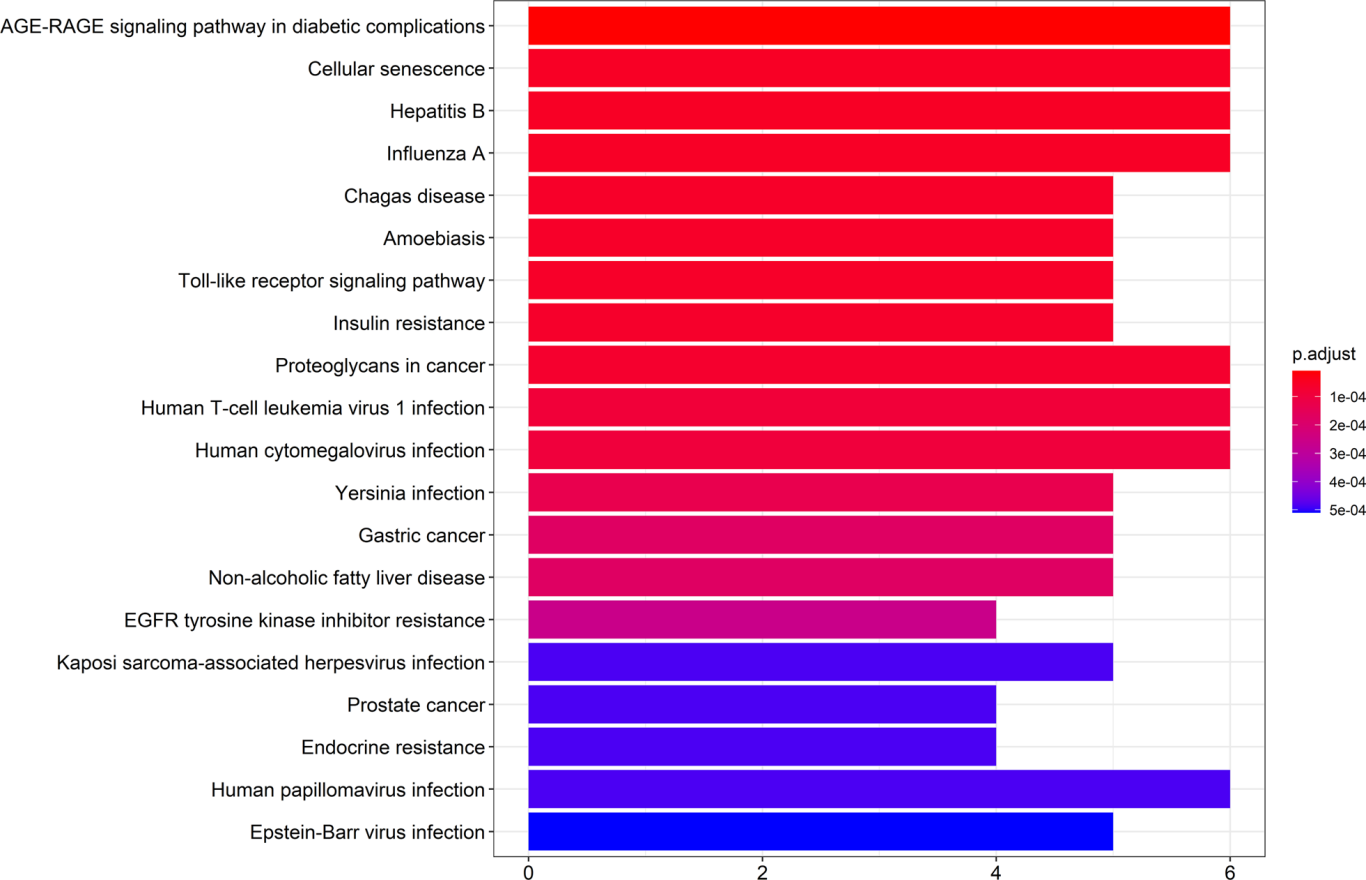
Histogram of KEGG functional enrichment analysis

**Fig. 10:**
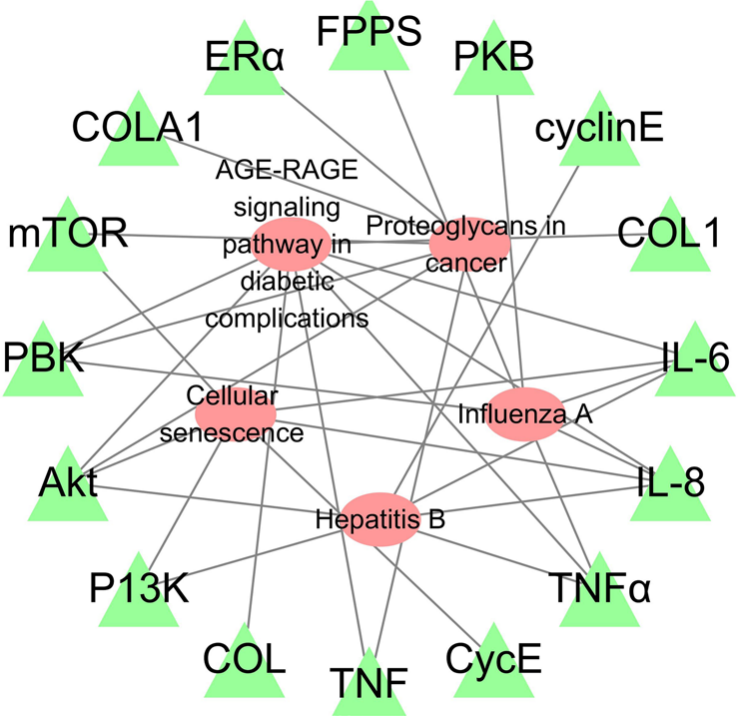
Key signaling pathways of overlapping drug-disease targets

We obtained the results of the KEGG enrichment analysis in Figs. 9 and 10. From these results, we concluded that the anti-OP IPRN is mainly involved in the PI3K-Akt signaling pathway, AGE-RAGE signaling pathway in diabetic complications, proteoglycans in cancer, TNF signaling pathway, and other biological pathways.

### Molecular docking

**Fig. 11:**
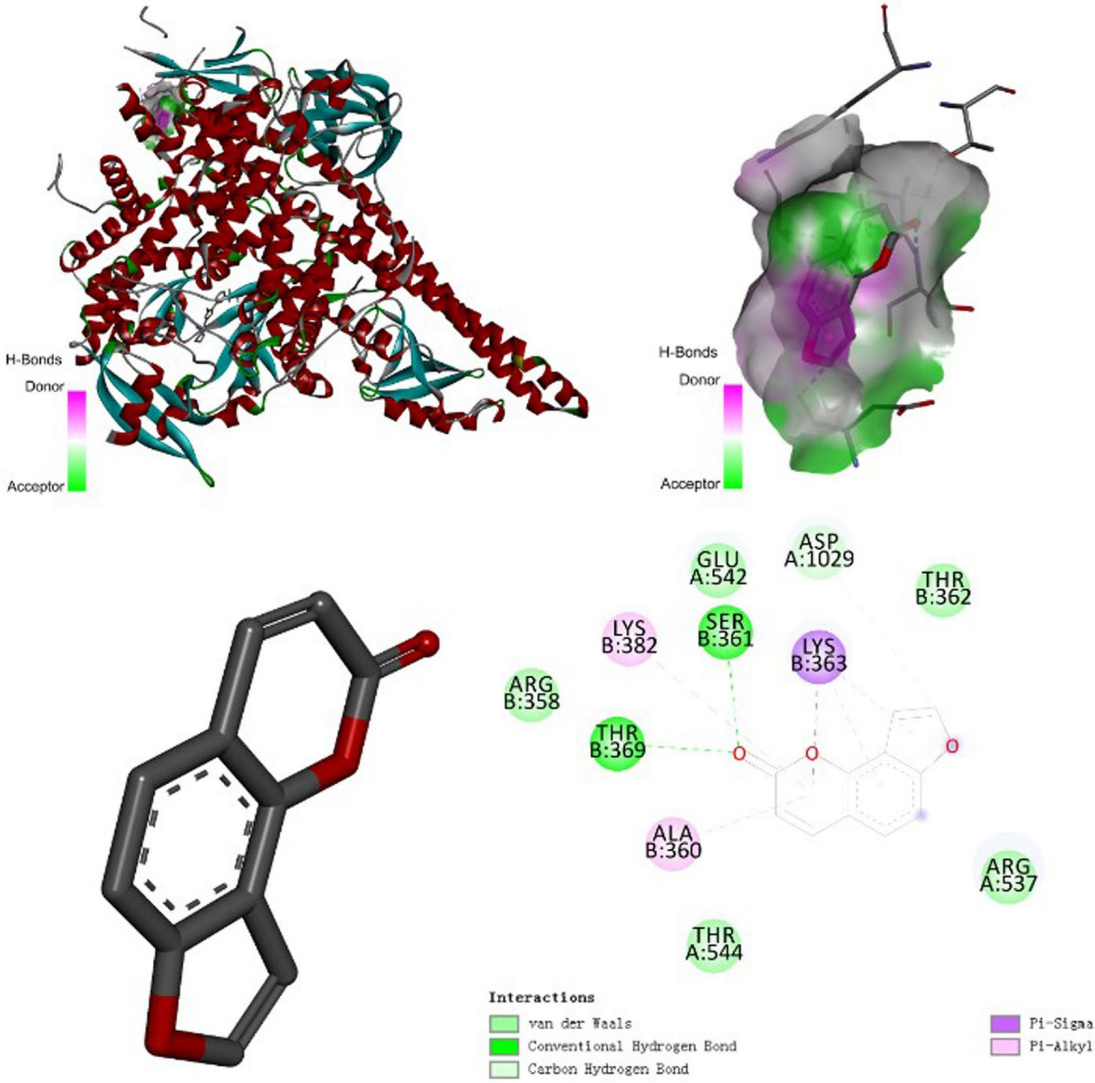
Schematic diagram of the molecular docking between isopsoralen and its target (PI3KCA)

**Fig. 12:**
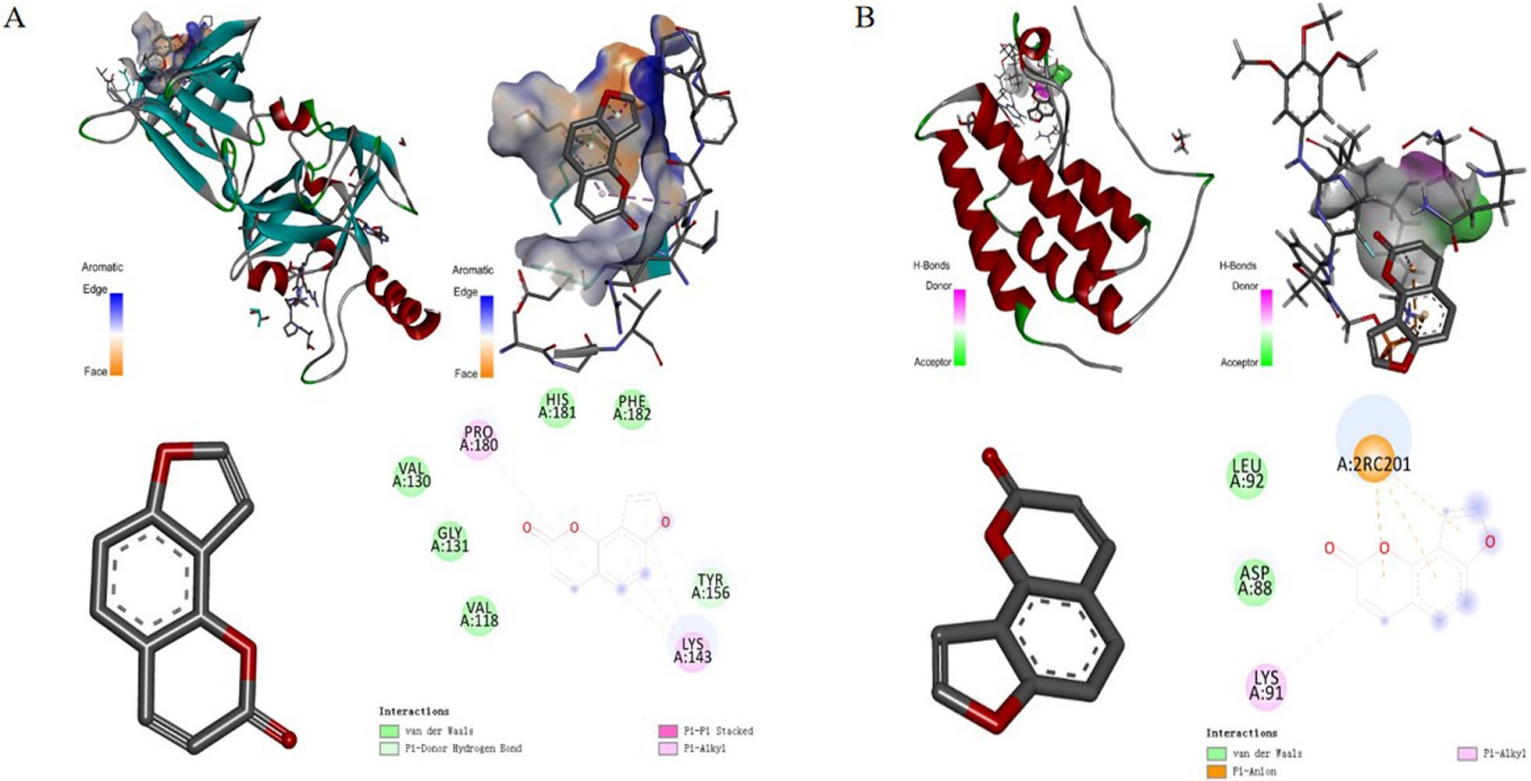
Schematic diagram of the molecular docking between isopsoralen and its target (**A**: AKT1; **B**: mTOR)

According to the final prediction results of network pharmacology and literature review, first, from the RSCB PDB database (http://www.rcsb.org/) download the AKT1 protein structure (PDB ID: 3OS5), PIK3CA protein structure (PDB ID: 4JPS) and mTOR protein structure (PDB ID: 4O75). Then we use AutoDock Vina 1.1.2 software to dock these target proteins (AKT1, PIK3CA and mTOR) with IPRN. The larger the absolute value of the Docking Score is, the stronger the bond between the compound and the target becomes, and the more stable the molecular conformation becomes. Therefore, we concluded that PIK3CA has the strongest binding force to IPRN, and its molecular structure is the most stable one (Fig. 11). The second is AKT1 (Fig. 12A), and the weakest one is mTOR (Fig. 12B). The binding energy of IPRN and the molecular docking of related targets are shown in Table 3.

**Table 3:** Molecular docking of Isopsoralen with related targets

Monomer component	AKT1	mTOR	PIK3CA
Isopsoralen	Binding energy/kcal·mol^−1^	Binding energy/kcal·mol^−1^	Binding energy/kcal·mol^−1^
	− 6.2	− 5.2	− 6.4
Amino acid name of docking score	VAL\GLY\PRO\HIS\PHE\TYR\LYS	LEU\ASP\LYS	THR\ARG\ALA\LYS\SER\GLU\ASP

### Molecular dynamics simulation

**Fig. 13:**
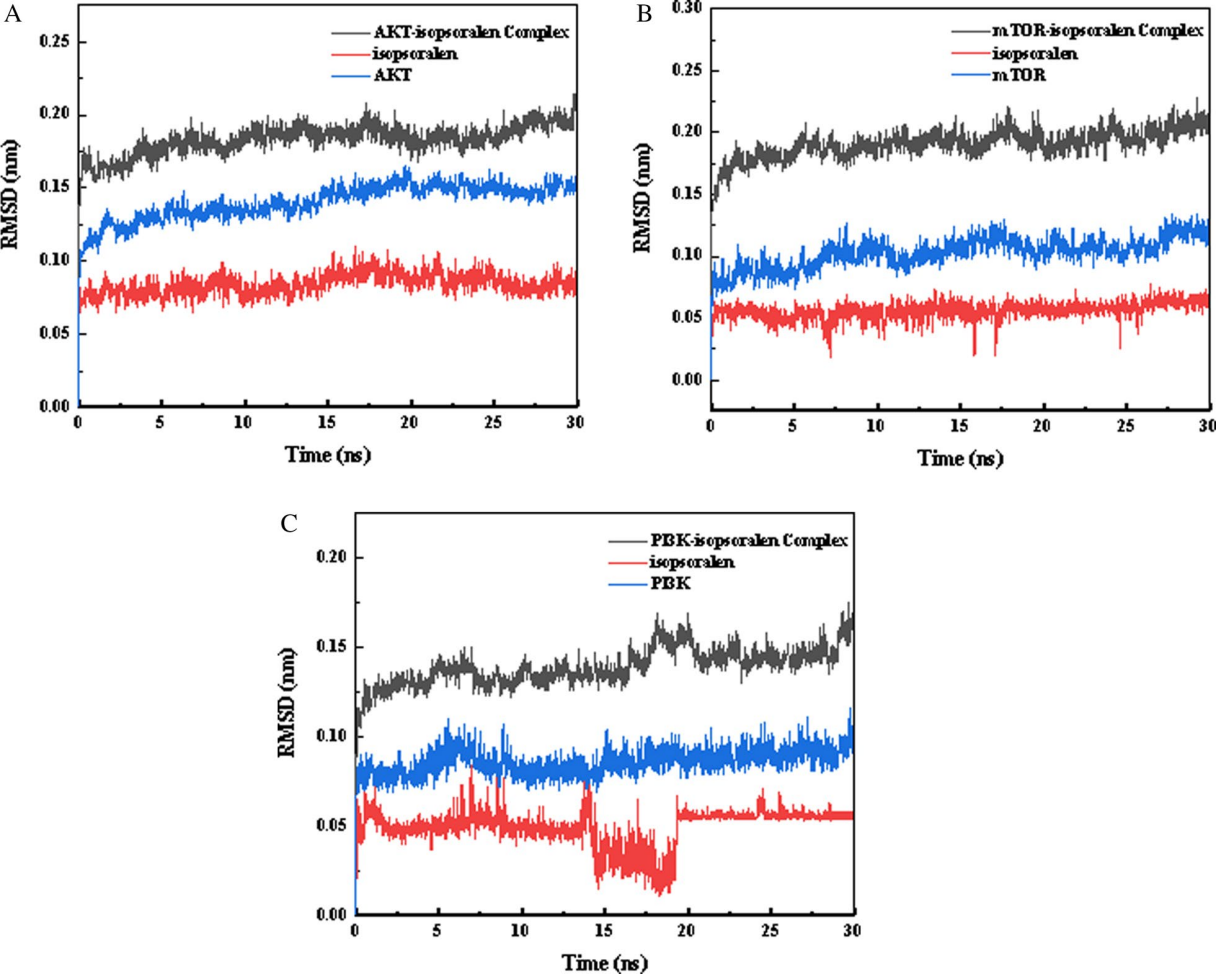
Root-mean-square deviation (RMSD) plots of AKT(3OS5)-IPRN (**A**), mTOR(4O75)-IPRN (**B**), PI3K(4JPS)-IPRN (**C**) during of molecular dynamics simulation

MD simulation provides insight into the stability of protein–ligand complexes. In this study, based on the docking results, PI3K(4JPS)-IPRN, AKT(3OS5)-IPRN, and mTOR(4O75)- IPRN were subjected to molecular dynamics simulation analyses for 100 ns to evaluate the motion, trajectory, structural features, binding potential, and conformational changes of molecules. The root means square deviation (RMSD) is a good measure of the conformational stability of protein and ligands and is a measure of the extent of deviation in the position of atoms from the starting position. A lower deviation indicates better conformational stability. The changes in RMSD values of the complexes were analyzed in this study. As shown in Fig. 13A, the RMSD of the AKT-IPRN composite system reached equilibrium after 10 ns. In the same way, the RMSD of the mTOR- IPRN composite system reached equilibrium after 7 ns (Fig. 13B), and the fluctuation value was small during the whole simulation process; Although the RMSD of PI3K- IPRN composite system fluctuated, it tended to be stable after 20 ns (Fig. 13C). This shows that the conformation of the protein does not change significantly after the combination of a small molecular ligand and protein, and the combination is relatively stable.

### Results of molecular experimental validation

#### Cell experiments

**Fig. 14:**
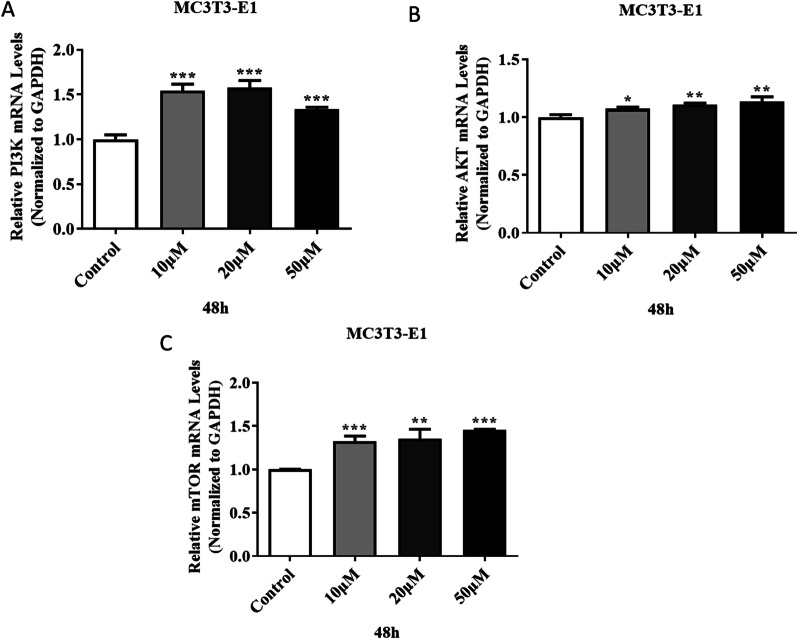
The expression of PI3K (**A**), AKT (**B**), mTOR (**C**) genes. *P < 0.05, **P < 0.01, ***P < 0.001

**Fig. 15:**
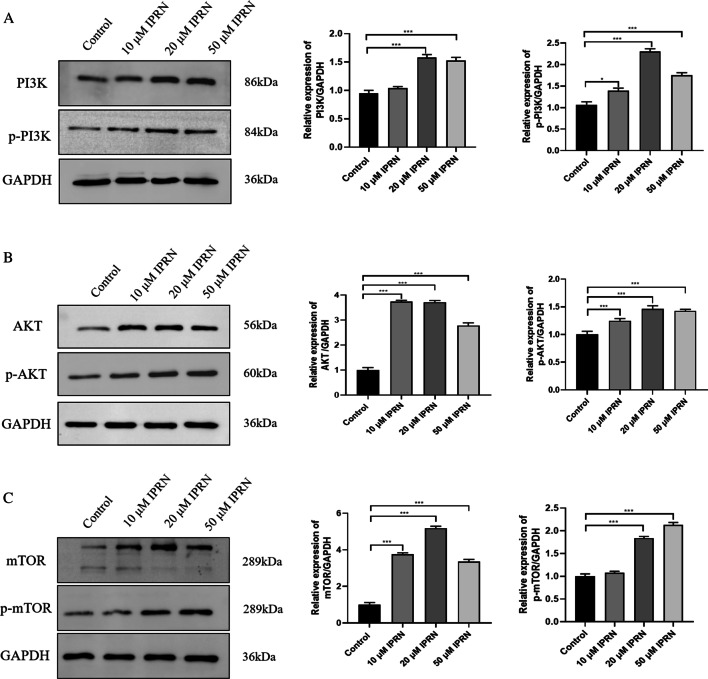
The expression of PI3K/p-PI3K (**A**), AKT/p-AKT (**B**) and mTOR/p-mTOR (**C**). *P < 0.05, **P < 0.01, ***P < 0.001

In the early stage of this experiment, by exploring the administration time, 24, 48, and 72 h were selected, and it was found that the difference in gene expression was more obvious when the drug was administered at 48 h. Therefore, 48 h was chosen as the experimental condition. Figure 14 shows the gene expression of the MC3T3-E1 cell line at 48 h and different drug concentrations. As can be seen from the figure, compared with the control group, 10, 20, and 50 μM concentrations in the 48 h group can significantly increase the expression level of PI3K, but with the increase of the dose, the effect of promoting the expression of PI3K does not increase significantly, and even appears downward trend (*P* < 0.001). In addition, compared with the control group, IPRN at concentrations of 10, 20, and 50 μM in the 48 h group could significantly increase the expression levels of AKT and mTOR, and it was positively correlated with the dose (*P* ˂ 0.05). More importantly, WB results showed that the expression of PI3K/p-PI3K, AKT/p-AKT, and mTOR/p-mTOR was significantly increased when 20 μM IPRN was added compared with the control group (Fig. 15A–C). In a word, the above results suggest that IPRN may affect the growth of MC3T3-E1 by participating in the PI3K-AKT-mTOR signaling pathway.

#### Animal experiments

**Fig. 16:**
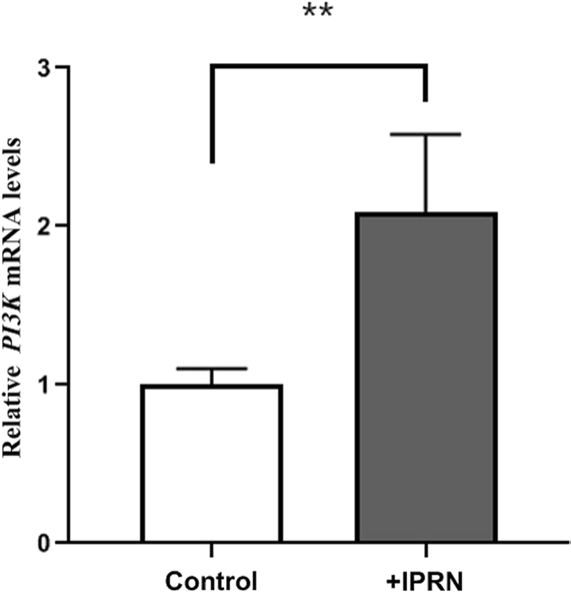
The expression of PI3K and β-actin. *P < 0.05, **P < 0.01, ***P < 0.001

Knee articular cartilage tissues were collected from rats in the experimental group and the control group, and qRT-PCR was used to detect related genes, and then the Ct values and expression levels of β-actin and PI3K in each group were obtained (Fig. 16). After the calculation of 2^−ΔΔCt^, they conformed to the normal distribution. The results showed that 40 mg/kg/time IPRN could increase the expression level of the PI3K gene in chondrocytes of SD rats after gavage for 7 days, which was significantly higher in the experimental group than that in the control group (*P* < 0.01). This result suggested that IPRN may exert an anti-OP effect by activating the PI3K gene.

## Discussion

OP is a degenerative disease characterized by decreased bone mass, degeneration of bone microarchitecture, and increased risk of bone fragility, which results in fractures and seriously endangers the health of middle-aged and elderly people [27]. At this stage, the side effects of Western medicine in the treatment of OP have caused considerable controversy and therefore, the researchers pay more attention to TCM which emphasizes the regulation of systemic function. As a medicinal ingredient, IPRN has potential advantages in the multi-targeted treatment of OP. Therefore, based on network pharmacology, this study systematically explored the pharmacological effects and potential targets of IPRN and discussed the potential molecular mechanism of IPRN in the treatment and prevention of OP through molecular experiments. The results of this study demonstrated that the anti-OP effect of IPRN is achieved through multiple targets and multiple pathways.

As we have learned, the bone tissue of the human body will be continuously updated with the progression of osteoblast-mediated bone formation and osteoclast-mediated bone resorption. In old age, bone resorption is significantly greater than formation, which causes bone loss. Previous studies have shown that IPRN can promote osteoblast differentiation and mineralization, increase calcium nodule levels and alkaline phosphatase (ALP) activity, and upregulate osteoblast markers including ALP, RUNX2, and COL1A1 [21]. Furthermore, IPRN can also alleviate oxidative stress-induced osteoblast injury through the Wnt/β-catenin signaling pathway [28]. The latest literature pointed out that the eight best differential genes, including AKT1, have a high diagnostic effect on the risk of OP [29]. In addition, tumor necrosis factor-α (TNF-α) can promote osteoclast differentiation to enhance bone resorption and inhibit osteoblast differentiation to impair bone formation [30]. In this study, a network diagram of IPRN-OP interacting proteins was constructed, with a total of 18 overlapping genes, including RUNX2, COL1A1, AKT1, and TNF. On the one hand, the results reflected that the best interaction point of IPRN was found by network pharmacology. On the other hand, it has been confirmed that IPRN can promote the expression of PI3K, AKT, and mTOR genes in MC3T3-E1 cells after 48 h. And in vivo experiments have also confirmed that 40 mg/kg/time IPRN can promote the PI3K gene expression in SD rat chondrocytes. These results expanded the idea of studying the specific pathogenesis of OP.

From another perspective, the anti-OP mechanism of prescriptions of TCM involves multiple regulatory pathways, such as PI3K/AKT/m TOR [31], Wnt/β-catenin [32], BMP/Smad [33], MAPK pathway [34], and so on. Based on the KEGG pathway enrichment analysis, we found that PIK3CA, AKT1, and mTOR were all enriched in the PI3K/AKT/mTOR signaling pathway. According to the literature, this pathway plays a crucial role in the pathological process of OP, and the activation of the PI3K/AKT/mTOR pathway can promote the differentiation of bone component cells including osteoblasts, chondrocytes, myoblasts, and adipocytes [35–37]; Naringenin exerts a protective effect on glucocorticoid-induced OP by regulating the PI3K/AKT/mTOR signaling pathway [38]. IGF-1 stimulates osteoblast precursors to form osteoblasts through the PI3K/AKT/mTOR pathway and promotes osteogenic differentiation [39]. In this study, the key genes of this pathway were verified in the follow-up experiments, and it was further concluded that IPRN may exert its anti-OP effect through the PI3K/AKT/mTOR pathway. In addition, the literature pointed out that the functional enrichment analysis of the targets of Liuwei Dihuang Pills in diabetic nephropathy-related OP found that the AGE-RAGE signaling pathway in diabetic complications was related to the main targets [40]. Similarly, this study enriched the same pathway by IPRN. The results suggested that IPRN has a positive effect in the treatment of diabetic nephropathy-related OP and can be used as a therapeutic drug.

Although this study has obtained the above results, it still has its shortcomings. First of all, the research on animal models of OP with TCM preparations is mainly to find new drugs or to further prove efficacy. Animal experiments in this study are relatively simple, just to verify whether the effect of IPRN on OP is obvious. Human OP is divided into primary and secondary, and animal models may not be completely consistent with the disease, so it is still necessary to improve and refine this aspect in subsequent research. Second, the verification of relevant protein expression is lacking in animal experiments. In the future, we are going to add WB, immunohistochemistry (IHC), and other methods to detect the expression of proteins. Despite the above shortcomings, our study still found that IPRN can make an anti-OP difference, which further complements the molecular mechanism of TCM in the treatment of OP.

## Conclusion

Based on network pharmacology, this study predicted the target genes of IPRN in the treatment of OP and preliminarily verified by in vitro and in vivo experiments that IPRN exerts an anti-OP effect by regulating the PI3K/AKT/mTOR pathway.
